# Comparing Bayesian Variable Selection to Lasso Approaches for Applications in Psychology

**DOI:** 10.1007/s11336-023-09914-9

**Published:** 2023-05-23

**Authors:** Sierra A. Bainter, Thomas G. McCauley, Mahmoud M. Fahmy, Zachary T. Goodman, Lauren B. Kupis, J. Sunil Rao

**Affiliations:** 1 Department of Psychology, University of Miami, 5665 Ponce de Leon Blvd, Coral Gables, FL 33146 USA; 2 Department of Psychology, University of California San Diego, San Diego, USA; 3 Department of Industrial Engineering, University of Miami, Coral Gables, USA; 4 Department of Psychiatry and Biobehavioral Sciences, University of California Los Angeles, Los Angeles, USA; 5 Division of Biostatistics, University of Miami, Coral Gables, USA

**Keywords:** Bayesian, regression, lasso, variable selection, penalization, shrinkage priors, stochastic search variable selection

## Abstract

In the current paper, we review existing tools for solving variable selection problems in psychology. Modern regularization methods such as lasso regression have recently been introduced in the field and are incorporated into popular methodologies, such as network analysis. However, several recognized limitations of lasso regularization may limit its suitability for psychological research. In this paper, we compare the properties of lasso approaches used for variable selection to Bayesian variable selection approaches. In particular we highlight advantages of stochastic search variable selection (SSVS), that make it well suited for variable selection applications in psychology. We demonstrate these advantages and contrast SSVS with lasso type penalization in an application to predict depression symptoms in a large sample and an accompanying simulation study. We investigate the effects of sample size, effect size, and patterns of correlation among predictors on rates of correct and false inclusion and bias in the estimates. SSVS as investigated here is reasonably computationally efficient and powerful to detect moderate effects in small sample sizes (or small effects in moderate sample sizes), while protecting against false inclusion and without over-penalizing true effects. We recommend SSVS as a flexible framework that is well-suited for the field, discuss limitations, and suggest directions for future development.

**Supplementary Information:**

The online version contains supplementary material available at 10.1007/s11336-023-09914-9.

Predictor selection is a common and complex problem in psychology. Which risk factors predict the development of autism? What psychosocial predictors relate to engagement in HIV care? What aspects of a relationship predict long-term success? The impact of predictor selection on the final model results is well known. This is exemplified, for example, by the fact that study preregistration requires specifying the model predictors, including which covariates may be included (van ’t Veer & Giner-Sorolla, 2016). In reality it may be difficult for many researchers to narrow down a set of predictors based purely on theory; researchers may also be unfamiliar with the potential pitfalls of both informal and automated (e.g. stepwise regression) variable selection methods.

Modern statistical methods developed for the dual purposes of regularization and variable selection have recently been introduced and used more widely within psychology, in particular the least absolute shrinkage and selection operator or lasso regression (Tibshirani, 1996). While lasso is an improvement over OLS regression in terms of prediction accuracy, variable selection accuracy can be inconsistent (Fan & Li, 2001; van Erp et al., 2019). In addition to the lasso, a wide variety of variable selection methods are available from both Bayesian and frequentist perspectives, all optimized to perform differently in terms of shrinkage, selection, and other criteria; however, the literature introducing and comparing different methods is sizeable and technical. A recent review shows that many regularization methods perform similarly in terms of prediction accuracy (van Erp et al., 2019) but differ in variable selection accuracy.

In the current paper, we review existing tools for solving variable selection problems in psychology with a focus on linear regression. We argue that Bayesian variable selection has advantages that make it well suited for researchers in psychology. We demonstrate these advantages and contrast Bayesian variable selection with classic and modern frequentist approaches. Our focus is primarily on variable selection accuracy, both in terms of false inclusion (Type I errors) and false exclusion (Type II errors) of predictors, as variable selection is a key inference for researchers in psychology. Our intention is to provide a non-technical and practical description of available methods, their limitations, and strengths for different research contexts. The results presented may also serve to inform power considerations for researchers designing studies, power being a key consideration for securing funding and for providing transparency in study planning and preregistration.

The remainder of this paper is organized as follows: in the next sections we review some key variable selection methods available in traditional and Bayesian frameworks. We compare these methods in the context of a motivating example and in a simulation study grounded by psychological applications. Finally, we review limitations and future directions.

## Traditional and Modern Variable Selection Methods

The linear regression model is a fundamental hypothesis testing framework used in the social sciences. That is, for $i =$ 1, …, *n* and a set of *p* predictors our model for the response variable *Y* is

1 $$\begin{aligned} y_{i}=\beta _0+\sum _{j=1}^p x_{ij} \beta _j +\epsilon _i, \quad \quad \epsilon _i \,\mathop {\sim }\limits ^{iid} N(0, \sigma ^2) \end{aligned}$$

with intercept $\beta _0$, regression coefficients $\beta _1,..., \beta _p$, and residual error variance $\sigma ^2$. Researchers must determine which predictors should be included in *x*. The choice of predictors may be completely pre-specified according to a specific research question (e.g. does the relationship between cognition and brain network connectivity differ by age, controlling for sex and head motion?). Frequently researchers may be interested in simplifying their model: narrowing the predictor set from a set of candidate predictors, a process called variable selection. For example, identifying risk factors of postpartum anxiety and depression (van der Zee-van den Berg et al., 2021), or identifying transdiagnostic factors that predict depression when controlling for demographic factors (Chen et al., 2021). Although researchers in psychology commonly encounter variable selection problems, many researchers remain unaware of suitable variable selection methods.

As noted by McNeish (2016), stepwise regression is still a prevalent practice in published psychological research, even though methodologists have long cautioned that stepwise regression approaches capitalize on sampling error, have poor replicability, and do not correctly identify the best predictor set of a given size (Henderson & Denison, 1989). Another common process for variable selection is to select predictors based on “univariable screening” or “prescreening” for significant *p* values (Babyak, 2004). Prescreening for significant bivariate relationships is problematic because these relationships are not guaranteed to translate to significant relationships in a multiple regression model, controlling for other (likely correlated) predictors. Univariable screening is akin to a forward selection approach in which insignificant predictors from the first step are not included in future steps, thus this strategy is even worse than stepwise modeling (Harrell, 2015). Any of these methods is expected to result in overfitting, poor generalizability, and misleading inferences (Thompson, 1995).

Once a model is specified, ordinary least squares (OLS) regression parameter estimates may be obtained that minimize the sum of squared errors, which we can write as

2 $$\begin{aligned} \hat{\beta }^{\hbox {OLS}}=\mathop {{\hbox {argmin}}}\limits _\beta \left\{ \sum _{i=1}^{N} \left( y_i - \beta _0 -\sum _{j=1}^{p} x_{ij}\beta _j\right) ^2\right\} . \end{aligned}$$

In contrast, the least absolute shrinkage and selection operator (lasso; Tibshirani, 1996) and other regularization methods add a penalty term which shrinks coefficient estimates towards zero. For the regularization to apply uniformly to all predictors regardless of scale, the predictor variables are standardized before estimation. Lasso estimates $\hat{\beta }$ by minimizing

3 $$\begin{aligned} \hat{\beta }^{\textrm{lasso}}=\mathop {{\textrm{argmin}}}\limits _\beta \left\{ \frac{1}{2}\sum _{i=1}^{N}\left( y_i - \beta _0 -\sum _{j=1}^{p}x_{ij}\beta _j\right) ^2+\lambda \sum _{j=1}^{p}\Big \vert \beta _j\Big \vert \right\} . \end{aligned}$$

Compared with the equation for $\hat{\beta }^{{OLS}}$, we can see that the lasso includes a penalty of the absolute sum of the coefficients weighted by a penalty term $\lambda$. The value for this parameter cannot be solved for analytically, and an optimal value is selected based on cross-validation or information criteria such as AIC or BIC. Cross-validation is used most often, because information criteria require computing degrees of freedom which is not straightforward to calculate with lasso (Zou et al., 2007). Cross-validation is done by splitting the data into *K* folds, for example 10, and then for all folds ($k =$ 1, …, *K*) estimating coefficients with lasso on $K-1$ folds of the data and using the estimates to predict values in the *k*th fold. Mean squared error or some other measure of fit is calculated for each fold for a particular value of $\lambda$. The two most common methods for selecting the optimal value of $\lambda$ are (1) selecting the value of $\lambda$ that minimizes the cross-validated error, and (2) selecting a value of $\lambda$ that is within one standard error of the minimum mean squared error value at the minimum $\lambda$. We will refer to these as the lasso(min) and the lasso(1SE) solutions. Lasso(min) has the attractive property of minimizing cross validation error but may not provide the desired level of shrinkage, whereas the lasso(1SE) solution can be used to arrive at a more conservative solution (i.e. more shrinkage, Hastie et al., 2009).

There are some attractive properties of the lasso which contribute to its widespread popularity^1^ in many disciplines. Bias introduced by regularization methods, including lasso, decreases variability in the parameter estimates and reduces overfitting. The lasso was developed to address limitations with two standard modifications to the OLS solution: subset selection methods and ridge regression. Whereas subset selection (e.g. stepwise regression) reduces model complexity but provides poor prediction accuracy, ridge regression is more stable in terms of prediction accuracy but does not reduce model complexity by setting any coefficients to zero. Because lasso sets some coefficients to zero, it performs regularization together with automatic variable selection. The optimal lasso solution can also be quickly obtained using efficient algorithms such as coordinate descent (Friedman et al., 2010).

However, as transformative as the lasso has been in statistics and beyond (Tibshirani, 2011), there are some well-documented limitations to lasso regression. Each limitation has motivated several extensions. For example, because lasso regression performs poorly when predictors are correlated—tending to select only one of a group of correlated predictors—the elastic net was developed to improve performance with correlated predictors (Zou & Hastie, 2005). The elastic net combines the lasso penalty on the sum of the absolute coefficients with the ridge penalty on the sum of the squared coefficients, as

4 $$\begin{aligned} \hat{\beta }^{\mathrm{elastic \,\, net}}=\mathop {{\textrm{argmin}}}\limits _\beta \left\{ \frac{1}{2}\sum _{i=1}^{N}\left( y_i - \beta _0 - \sum _{j=1}^{p}x_{ij}\beta _j\right) ^2+\lambda _{1}\sum _{j=1}^{p}\Big \vert \beta _j\Big \vert +\lambda _2 \sum _{j=1}^{p}\beta _j^2\right\} . \end{aligned}$$

Further, the lasso can estimate at most *n* nonzero coefficients, which is problematic when $n > p$, and many methods have been developed that better scale to accommodate high dimensional data (e.g. Ishwaran & Rao, 2014, 2005b; Zou & Hastie, 2005). Another limitation is that the lasso applies a single penalty term across all coefficients, thereby overshrinking meaningful large coefficients, and various modern methods aim to correct this bias in the lasso by adaptively shrinking coefficients (Fan & Li, 2001). Finally, the lasso will only consistently select the true predictors under specific and nontrivial conditions, meaning asymptotically the procedure may not recover the true model with probability leading to 1. The adaptive lasso is one alternative developed for consistent variable selection (Zou, 2006), defined as

5 $$\begin{aligned} \hat{\beta }^{\textrm{adaptive}}=\mathop {{\textrm{argmin}}}\limits _\beta \left\{ \frac{1}{2}\sum _{i=1}^{N}\left( y_i - \beta _0- \sum _{j=1}^{p}x_{ij}\beta _j\right) ^2+\lambda \sum _{j=1}^{p}\hat{w}_j\Big \vert \beta _j\Big \vert \right\} \end{aligned}$$

which introduces a vector of weights **w** to assign a different penalty to each coefficient. Taken together, this literature suggests that the lasso may not be a suitable variable selection method for psychological applications, however choosing among the array of alternatives can be overwhelming given that the statistical literature is specialized, technical, and fast-growing.

All these properties: regularization, variable selection consistency, performance with correlated predictors, and ability to scale when the number of predictors exceeds the sample size, are important in some research contexts, but arguably some are more critical for most applications in psychology. Consistent variable selection (*which* predictors meaningfully relate to the outcome?) is a focal concern. Bias in coefficient estimates is also of central importance. It is also essential to consider variable selection performance when predictors are correlated, as we generally expect in psychology. Perhaps less concerning for many researchers in psychology is the constraint that the number of selected predictors cannot be larger than the sample size. This constraint is appropriate when theory can guide the selection of candidate predictors.

While adoption of lasso and related methodologies is not widespread within psychology, some psychological scientists have begun utilizing lasso regularization in a variety of contexts and applications. These include identifying predictors of non-suicidal self-injury (Ammerman et al., 2018), risk for developing psychosis (Moore et al., 2021), and psychosocial consequences of the COVID-19 pandemic (Zhu et al., 2021). Elastic net regularization has been used in psychological domains as well, including to examine the predictive power of personality traits (Seeboth & Mõttus, 2018), alexithymia and affect labeling in emotional scenarios (Aaron et al., 2018), and antidepressant treatment outcomes (Iniesta et al., 2016). Both lasso and elastic net methodologies have been implemented in neuroscience, to establish patterns of neural coactivation (Carroll et al., 2009; Kauttonen et al., 2015) as well as to examine brain-behavior correlates of psychopathology (e.g., van Rooij et al., 2018) and cognitive impairment (e.g., Fagerholm et al., 2015). Besides regression modeling, lasso regularization is used in psychology embedded within more complex methodologies, such as network analysis (Epskamp & Fried, 2018), structural equation modeling (Jacobucci et al., 2019), and automated detection of differential item functioning (Bauer et al., 2020; Magis et al., 2015).

To summarize, it is crucial to consider the performance of variable selection methods for applications in psychology, and the automatic variable selection and shrinkage imposed by the lasso has significant limitations that should be considered and weighed against alternatives. Whereas lasso-type estimators achieve variable selection as a byproduct of regularization, the variable selection problem can be approached more directly in a Bayesian framework. We next consider variable selection methods from a Bayesian perspective.

## Bayesian Variable Selection Methods

A Bayesian variable selection framework offers added flexibility for how to appropriately shrink estimates and perform variable selection. Whereas the penalized likelihood approaches reviewed in the previous section *jointly* perform selection and regularization by adding a penalty term to the minimization of the sum of squared residuals, these aspects can be addressed more independently and directly in a Bayesian framework. The Bayesian perspective includes a prior distribution for each parameter in the model, which contains information about uncertain parameter estimates, that is combined with the probability distribution of new data to yield the posterior distribution, which is used for inference. In Bayesian penalized regression, the penalty parameter $\lambda$ is a parameter in the prior for the regression coefficients $\beta _{j}$. Bayesian penalized regression models are estimated using Markov Chain Monte Carlo (MCMC) sampling rather than optimization. MCMC estimation can be thought of as Monte Carlo integration using Markov chains; these algorithms provide a flexible approach to systematically sample from the target posterior distribution at the cost of increased computational time relative to standard optimization procedures. parameter estimates, which may be relatively concentrated or diffuse, flexibly allowing for regularization.

The penalized regression solutions described in the previous section can be obtained in the Bayesian framework by using specific prior distributions combined with the posterior mode estimate. For example, Park and Casella (2008) showed that the lasso estimates can be obtained by placing independent Laplace (i.e. double-exponential) priors on the regression coefficients:

6 $$\begin{aligned} \beta |\sigma ^2=\frac{\lambda }{2\sqrt{\sigma ^2}}e^{-\lambda |\beta _j|/\sqrt{\sigma ^2}} \end{aligned}$$

Similarly, prior specifications can be used to obtain the ridge (Hsiang, 1975), elastic net (Li & Lin, 2010), and adaptive lasso (Leng et al., 2014) penalized estimates as Bayesian posterior mode estimates. As in the frequentist literature, each of these modeling choices optimize various tradeoffs between regularization and variable selection for particular characteristics of the data.

Although most prior specifications can be considered regularizing to some extent, fully Bayesian variable selection can be used to directly estimate the posterior probability that each predictor should be included in the model. Fully Bayesian variable selection is accomplished by adopting what is called a spike-and-slab prior. This name refers to priors formulated as a discrete mixture of a “spike” that is concentrated around zero and a diffuse “slab” of nonzero values. A central aspect of this approach is the addition of an indicator variable $\delta _{\, j}$ for each predictor which allows for switching between the spike and the slab mixture components (where $\delta _{\, j} =$ 1 indicates that predictor *j* is included, and $\delta _{\, j} =$ 0 indicates absence of predictor *j*) as in (7).

7 $$\begin{aligned} y_i=\sum _{j=1}^{p}\delta _{j} x_{ij}\beta _{j}+\epsilon _{i} \end{aligned}$$

Some of the prior distributions mentioned above (e.g. Bayesian lasso) may be parameterized as *continuous* (rather than discrete) mixtures and approximate a spike-and-slab distribution in terms of shape, but the defining feature of spike-and-slab Bayesian variable selection approaches is the ability to estimate the probability that each coefficient is zero versus not equal to zero. George and McCulloch (1993) first introduced the term stochastic search variable selection (SSVS) to describe their formulation, and here we use the term SSVS to refer generally to Bayesian variable selection procedures with a discrete mixture prior and using MCMC estimation.

Specific Bayesian variable selection methods differ in details of how the prior distribution, model, and MCMC sampler are specified, but in many standard cases the methods produce highly similar results (O’Hara & Sillanpää, 2009). In terms of the prior, the spike is usually formulated as either a point mass at zero (Mitchell & Beauchamp, 1988), which sets coefficients in this component exactly to zero, or a normal component tightly concentrated around zero (George & Mcculloch, 1993). We will demonstrate the former approach in this paper, also corresponding to the default specifications in the SSVS R package (Bainter et al., 2022). The prior for $\beta _{j}$ is

8 $$\begin{aligned} \begin{array}{l} \beta _{j} \vert \delta _{j} \sim N(0,10), \\ \delta _{j} \sim \hbox {Bernoulli}(\pi _{j} ) \\ \end{array} \end{aligned}$$

with the prior probability of inclusion set at $\pi _{\, j} =.5$ for each predictor, reflecting a prior belief that 1/2 of the predictors should be included. Noninformative priors are included for the nuisance parameters: $\beta _{0}$ ~$U[-\infty ,\infty$] and 1/$\sigma ^{2}$ ~Gamma($a =.01, b =.01$). Just as for the lasso approaches, for the prior to apply to all predictors regardless of scale, the predictor variables are standardized before estimation.

After MCMC estimation, the posterior summary of most importance is the marginal inclusion probability (MIP) for each predictor, calculated as the proportion of MCMC samples with $\delta _{j}=1$ (i.e. the proportion of samples in which $\delta _{j}$ is included). The MIPs can be used to approximate the posterior probability that the predictor should be included in the model. These continuous quantities provide an intuitive index of the importance of predictors, specifically which predictors are reliably related to the outcome, accounting for uncertainty in which other variables are included (i.e. “controlled for”) in the model. Whereas the lasso approaches perform automatic variable selection (setting coefficients to zero), the MIPs obtained using SSVS are used to inform variable selection. For example, if the prior inclusion probability for each predictor is .5, the model which includes all predictors with MIPs above .5 is termed the median model; this cutoff has been shown to be optimal for uncorrelated predictors under certain conditions (Barbieri & Berger, 2004). However, there are drawbacks to automatically relying on any statistical threshold, and it is also informative to graphically inspect the pattern of MIPs, as we will show in our motivating example in the next section. The marginal distributions of the coefficients $\beta _{j}$ may also be examined, which provide model averaged estimates of the coefficients. However, the model averaged estimates of coefficients may be challenging to interpret, because the interpretation of coefficients may depend on which variables are included in the model (Forte et al., 2018).

Limitations and properties of Bayesian variable selection methods should also be considered. Typical SSVS approaches require proper specification of the prior distributions for the parameter estimates and on the model space, posterior threshold choice, and efficient MCMC algorithms to estimate posterior probabilities. Results can be sensitive to each choice. Prior specification becomes more challenging in high dimensional models (Ishwaran & Rao, 2005a), and marginal inclusion probabilities may be difficult to estimate in high collinearity designs. However, posterior consistency arguments have been developed under certain identifiability conditions (e.g., Liang, Song, & Yu, 2013). Different MCMC methods for Bayesian variable selection have been developed to optimize posterior exploration, mixing, computational efficiency, and ability to scale to large data sets (O’Hara & Sillanpää, 2009).

Relative to frequentist regularization and variable selection approaches, Bayesian approaches can be used to directly address variable selection and provide quantitative information about variable performance, while still performing similarly in terms of prediction error (Porwal & Raftery, 2022; Viallefont et al., 2001). Van Erp et al. (2019) compared the variable selection accuracy and prediction error of a survey of Bayesian penalized regression methods. Their study was focused broadly on priors for regularization, including Bayesian lasso and elastic net lasso, but they also included a formulation for spike-and-slab Bayesian variable selection. For conditions with $p < n$, they found small differences among methods in terms prediction accuracy. They found larger differences with regards to variable selection accuracy, illustrating a trade-off between correct and false inclusion rates with no single method performing best across conditions. While Van Erp et al. (2019) highlighted differences among Bayesian regularization methods (including SSVS) in a select set of conditions, they did not systematically vary factors such as effect size, sample size, or the correlation structure among predictors that would help guide researchers in psychology. Bainter et al. (2020) compared SSVS to lasso regression in a targeted set of conditions based on a real data example, varying the sample size and reliability of the outcome variable. They found SSVS resulted in more stable selection of predictors in comparison with lasso, and the number of predictors selected using lasso varied widely by condition. Their design also did not vary the pattern, size, or number of true effects and did not examine false inclusion or correct inclusion rates.

Many key questions remain. How well do Bayesian variable selection methods correctly identify important predictors in a variety of conditions representative of psychological applications? How do factors such as the sample size, size of effects and pattern of effects, and correlations among predictors impact performance? Are simple SSVS algorithms computationally sufficient for moderately sized problems, or are specialized algorithms needed? And how does SSVS compare with lasso and its variants in these conditions?

## Motivating Example: Predicting Depression Symptoms

To illustrate the differences between lasso and SSVS, we consider a motivating example to identify predictors of depression across a range of both self-report and brain-based correlates of psychopathology. Given a wide range of potentially correlated predictors implicated by psychological theory and the literature, the motivating research question is to identify which factors are most important when simultaneously considering factors across domains.

Multiple conceptual models have been proposed to describe psychosocial processes associated with depressive symptoms. While not an exhaustive list, theorized constructs have included temperament and personality characteristics (Kudo et al., 2016; Scheier & Carver, 1992), emotional dysregulation and impulsivity (Dekker & Johnson, 2018; Johnson, Elliot, & Carver, 2020), disrupted sleep (Blake, Trinder, & Allen, 2018; Pandi-Perumal et al., 2020), and risk-taking behavior (Soleimani et al., 2017; Telzer et al., 2014). Increased availability of neuroimaging modalities has also led to a proliferation of research investigating brain-based correlates of psychopathology (Hamilton, Chen, & Gotlib, 2013). Disruptions in the integrity of three large-scale functional brain networks have been implicated in depressive symptomatology (Fischer, Keller, & Etkin, 2016; Menon, 2011; Mulders et al., 2015). These networks are commonly referred to as the *default mode network*, active in self-referential processes; the *central executive network*, responsible for maintaining and manipulating information in working memory; and the *salience network*, involved in attentional capture of relevant stimuli.

We set out to determine the most consistent predictors of depressive symptoms in a normative adult population drawn from the Nathan Kline Institute Rockland Sample, a large publicly-available lifespan sample with a rich array of assessments (Nooner et al., 2012). The sample included $N = 454$ adults aged 18 and above ($M = 37.5, \text {SD} = 14.1$) with data available for all measures. Depression symptom severity was measured via the Beck Depression Inventory—II (BDI-II; Beck, Steer, & Brown, 1996).

### Candidate Predictors

The full list of candidate self-report predictors and selected brain connectivity measures is shown in Table 1. A total of 23 self-report measures, spanning psychological traits, health behaviors, age, and sex were included as candidate predictors. Psychological measures of personality, empathy, impulsivity and risk-taking were four temperament subscales of the Adult Temperament Questionnaire (Evans & Rothbart, 2007), four empathy subscales of the Interpersonal Reactivity Index (Davis, 1983), five subscales of the Domain-Specific Risk-Taking scale (Blais & Weber, 2006), and five subscales of the UPPS-P Impulsive Behavior Scale (Whiteside & Lynam, 2001). To measure health behaviors, total scores were included drawn from the Pittsburgh Sleep Quality Index (Buysse et al., 1989), the Fagerstrom Test for Nicotine Dependence (Heatherton et al., 1991), and the International Physical Activity Questionnaire (Craig et al., 2003).

**Table 1:** Self-report and selected brain connectivity measures and coefficients—depression example.

Domain	Predictor	SSVS			Lasso (min)	Lasso (BIC)	Lasso (1SE)	Adaptive lasso	Elastic net
		MIP	Avg b	Avg b$\ne$ 0					
Demographic	Age	0.09							$-$ 0.00
	Sex (ref$=$female)	0.12			$-$ 0.02				$-$ 0.08
Personality	ATQ-Negative Affect	0.99	1.50	1.51	1.32	1.20	1.47	1.30	1.29
	ATQ-Extraversion	0.57	$-$ 0.40	$-$ 0.71	$-$ 0.55	$-$ 0.75		$-$ 0.60	$-$ 0.65
	ATQ-Orienting Sensitivity	0.71	0.55	0.77	0.62	0.84	0.07	0.69	0.84
	ATQ-Effortful Control	0.13							
	IPRI-Perspective-taking	0.09							$-$ 0.00
	IPRI-Fantasy	0.14							$-$ 0.23
	IPRI-Empathetic Concern	0.10							0.10
	IPRI-Personal Distress	0.44			0.53	0.59	0.00	0.51	0.63
	DOSP-Ethical Risk	0.32			0.35	0.56		0.38	0.47
	DOSP-Recreational Risk	0.16			0.10				0.31
	DOSP-Social Risk	0.09							$-$ 0.11
	DOSP-Financial Risk	0.08							
	DOSP-Health/Safety Risk	0.10							
	UPPS-Negative Urgency	1.00	1.47	1.48	1.17	1.38	0.66	0.10	1.32

	Predictor	SSVS			Lasso (min)	Lasso (BIC)	Lasso (1SE)	Adaptive lasso	Elastic net
Domain		MIP	Avg b	Avg b $\ne$ 0					
	UPPS-Premeditation	0.90	$-$ 0.92	$-$ 1.00	$-$ 0.56	$-$ 0.90		$-$ 0.75	$-$ 0.70
	UPPS-(Lack of) Perseverance	0.88	0.95	1.03	0.62	0.85	0.00	0.74	0.72
	UPPS-Sensation Seeking	0.13							0.01
	UPPS-Positive Urgnecy	0.26							$-$ 0.22
Health behaviors	PSQI-Sleep Quality	1.00	2.30	2.31	2.16	2.21	1.58	2.24	2.14
	IPAQ-Physical Activity	0.10			$-$ 0.02				$-$ 0.18
	FTND-Nicotine	0.30			0.34	0.52	0.03	0.32	0.51
Brain network	ACC_rDLPFC	0.18			$-$ 0.02				$-$ 0.35
connectivity	ACC_rFIC	0.29			$-$ 0.28	$-$ 0.49	$-$ 0.01	$-$ 0.17	$-$ 0.40
	ACC_rPPC	0.11							0.10
	ACC_VMPFC	0.17							0.23
	rDLPFC_rFIC	0.17							0.22
	rDLFPC_rPPC	0.12			0.06				0.19
	rDLPFC_vmPFC	0.18							$-$ 0.20
	rFIC_vmPFC	**0.78**	1.13	1.41	0.46	0.63	0.02	0.35	0.81
	rFIC_rPPC	0.31			0.20		< 0.01		0.36
	rFIC_PCC	**0.67**	$-$ 0.89	$-$ 1.23	$-$ 0.20				$-$ 0.75
	rPPC_vmPFC	0.15			0.06				0.17

MIP, Marginal Inclusion Probabilities; Avg *b*and* Avg b* $\ne$* 0 *are model averaged estimates from SSVS inclusive of and excluding zero values, respectively.

**Fig. 1:**
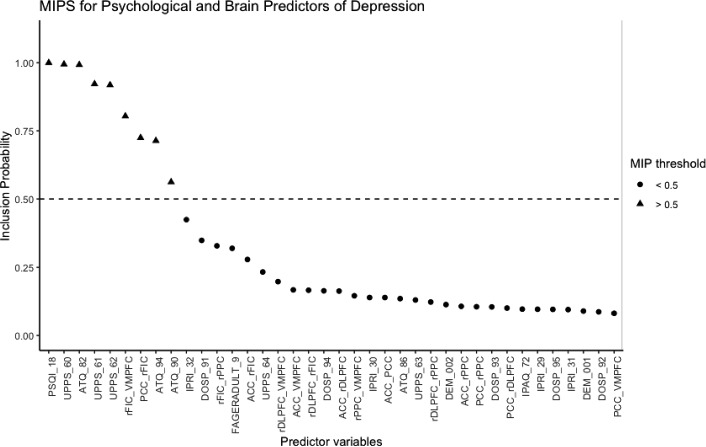
Pattern of Marginal Inclusion Probabilities from SSVS for motivating example data.

Additionally, 15 functional brain connectivity metrics were included as candidate predictors, for a total of 38 candidate predictors. Brain connectivity metrics were included to represent the functional connectivity of regions of interest within and between the default mode, executive control, and salience networks. Two regions were selected to represent each large-scale brain network: the anterior cingulate cortex (ACC) and right fronto-insular cortex (rFIC) represent the salience network, the right dorsolateral prefrontal cortex (rDLPFC) and right posterior parietal cortex (rPPC) from the central executive network, and the ventromedial prefrontal cortex (vmPFC) and posterior cingulate cortex (PCC) from the default mode network. Connectivity was calculated as the correlation between each region across a 10-minute resting-state functional magnetic resonance imaging (rs-fMRI) scan. Complete details of the preprocessing pipeline are reported elsewhere (Goodman et al., 2021).

### Variable Selection Methods Compared

Results were obtained in R using functions for SSVS, lasso, adaptive lasso, and the elastic net lasso. SSVS results were obtained using the ssvs() function from the SSVS R package (Bainter et al., 2022), which performs Gibbs sampling of the posterior distribution according to the point mass prior specification described in Sect. 2. We used the default prior specification and number of MCMC samples (20,000 draws with the first 5000 discarded as warm-up). Thus, we obtained posterior summaries of the MIPs and regression coefficients for each parameter averaged over 15,000 MCMC draws. For all lasso methods, we obtained solutions using 10-fold cross-validation. Lasso estimates using both the lasso(min) and the lasso(1SE) criterion were obtained using the cv.glmnet() function from the glmnet package (Friedman et al., 2021). Adaptive lasso estimates were obtained using custom code according to the algorithm described by Zou (2006). The elastic net solution was obtained using the caret package (Kuhn et al., 2022). Finally, as an additional comparison suggested by a reviewer, we refit candidate models identified from the lasso and selected the best based on BIC. Specifically, we refit the models identified by the lasso(min) criterion, the lasso(1SE) criterion, and candidate models between these two solutions. This solution is labeled lasso(BIC), however note the estimates are from the unregularized, refitted model chosen based on BIC.

### Results and Discussion for Motivating Example

Table 1 shows the predictors selected by each method and estimated coefficients. All results are based on standardized predictors. The pattern of MIPs from SSVS is displayed in Fig. 1. For SSVS, there were nine predictors with MIPS > .5, and the corresponding model averaged coefficient estimates are shown for predictors with MIPs above this threshold. Two sets of SSVS estimates are shown: average estimates including zero values and average estimates conditioned on inclusion (i.e. only non-zero values), which are less regularized. Each of the selected predictors explain unique variability in depressive symptoms while controlling for uncertainty with respect to which other variables are included in the model.

Based on this model, several psychological and neuroimaging metrics were consistently selected predictors of depressive symptoms. Total scores on the PSQI emerged as the most selected predictor and was included in 100% of models, indicating higher sleep dysregulation was consistently associated with greater depressive symptom severity. Additionally, three components of impulsivity had high MIPs. Negative Urgency, representing impulsivity arising from negative affective states, and (Lack of) Perseverance, representing a tendency to avoid or quit difficult tasks, were predictive of greater depressive symptoms. Premeditation refers to difficulty when evaluating the consequences of behavior and was predictive of lower depressive symptoms. Three scales of temperament from the ATQ were also selected. Negative Affect and Orienting Sensitivity, indicating low mood and a sensitivity to be influenced by environmental stimuli of mild valence, respectively, were associated with higher depressive symptoms, whereas Extraversion was associated with lower depressive symptoms. Two functional connectivity metrics were identified as consistent predictors by SSVS, both of which implicated connectivity between the FIC, a node of the salience network, and key nodes in the default mode network. Between-network hypoconnectivity across nodes of the salience and default mode networks, as indicated by connectivity between the FIC and PCC, was associated with greater depressive symptoms. Additionally, hyperconnectivity between the FIC and vmPFC was also associated with greater depressive symptom severity. Insular hypoconnectivity has been highlighted as a possible indicator of salience network dysfunction (Manoliu et al., 2014). The salience network modulates activation of other networks in response to perception of salient stimuli (Menon & Uddin, 2010), and is believed to be compromised in several psychopathologies (Menon, 2011).

The lasso solutions tended to select more predictors than SSVS, with varying degrees of regularization for the parameter estimates. The lasso(min) criterion included more than twice as many predictors as SSVS, 20, but the coefficient estimates tended to be smaller. In terms of substantive conclusions, the lasso(min) solution would imply a more complex model of unique predictors for depressive symptoms, each with weaker effects. With the exception of the elastic net, the models identified by SSVS and the other lasso variations all contain subsets of the 20 predictors selected by the lasso(min) criterion. The lasso(1SE) solution included 10 predictors, with markedly different estimates resulting from the further regularization only three are larger than .1 in absolute value.

The most consistency was seen between the lasso(BIC) and adaptive lasso solutions. Using the BIC criterion to select the best among candidate solutions identified by lasso, the selected model included 12 predictors. The estimates from this model are unregularized and generally larger in magnitude compared to the lasso(min) solution. The adaptive lasso solution included the same set of 12 predictors as lasso(BIC), and the magnitude of the nonzero coefficients is generally similar to those from lasso(min). These solutions contained several predictors not included in the SSVS model. Measures of empathy (personal distress), risk-taking (ethical risk-taking), and nicotine dependence were included and positively associated with depression. Hypoconnectivity between nodes of the salience network, the ACC and rFIC was also included with these lasso solutions. It is interesting to note that these predictors had intermediate MIPs, between about .3 and .5. Only one predictor-functional connectivity between the rFIC and PCC-was included in the SSVS solution but excluded by these lasso solutions.

Finally, the elastic net solution included the most predictors, 31 of the 38 total candidate predictors. This pattern of results summarizes the general behavior of each variable selection method, which we will compare more in depth using a simulation study in the next section.

## Simulation Study

To compare the performance of Bayesian variable selection to lasso type regularization, we simulated data from a variety of conditions realistic for psychological research. All code to perform the simulation is available on http://osf.io/5vp3k/.

**Table 2:** Simulation design.

Condition	$\rho _{xx}$	$\beta$
Independent	0.0	(1.5, .9, .9, .3, .3, 1.5, .9, .9, .3, .3, 0, ... , 0)
Mixed true effects	0.4	(1.5, .9, .9, .3, .3, 1.5, .9, .9, .3, .3, 0, ... , 0)
	0.8	
Clustered true effects	0.4	(1.5, 0, 0, 0, 0, .9, 0, 0, 0, 0, .9, 0, ... , 0)
	0.8	

$\rho _{xx = }$ (exchangeable) correlation among blocks of predictors; $\beta$ $=$ vector of standardized regression coefficients for small, medium, large, and null effects. Note that for conditions with nonzero correlation among predictors the correlation structure was block diagonal (BD) with five correlated predictors per block.

### Manipulated Factors and Data Generation Procedure

The simulation conditions are summarized in Table 2. The sample sizes examined were $n =$ 100 and 400, reflecting typical sample sizes and allowing us to examine how results varied by sample size. The number of candidate predictors was $p =$ 50. In each of the data generating conditions, 10 predictors had true effects: four small, four medium, and two large effects with standardized regression coefficients $\beta =.1,.3,.5$. Finally, the pattern of correlations among the predictors was varied. For a baseline condition, **X** was generated from a multivariate normal distribution with diagonal variances equal to 1 and all pairwise correlations equal to 0. The vector of coefficients contained true effects for the first 10 predictors, $\beta ' = (1.5,.9,.9,.3,.3, 1.5,.9,.9,.3,.3, 0, \ldots , 0$), and the response vector *Y* was drawn from $N_{n}$(**X** $\beta$, $\sigma ^{2}I)$, with $\sigma ^{2} =1$ resulting in four small, four medium, and two large effects with standardized regression coefficients $\beta =.1,.3,.5$.

To simulate conditions with clusters of correlated predictors, a set of conditions was included with predictors simulated from a multivariate normal distribution with block diagonal covariances. Sets of five predictors were correlated $\sigma =.4$ in a moderate condition and $\sigma =.8$ in a high correlation condition for clusters of predictors. Finally, the pattern of true effects was varied so that the true effects were either correlated or uncorrelated. For correlated true effects, the specification of $\beta$ was the same as above so that true effects were from two clusters of correlated predictors. For the condition with correlated predictors but uncorrelated true effects, we rearranged the 10 true effects so that every fifth coefficient was nonzero, $\beta ' = (1.5, 0, 0, 0, 0,.9, 0, \ldots , 0)$. Note that although the same regression coefficients were used to simulate data for conditions with correlated and uncorrelated predictors, the resulting effects are smaller for the conditions with clusters of correlated predictors with true effects. Simulations were conducted using *R* statistical software. For each condition 500 replications were generated.

### Variable Selection Methods

For each replication, results were obtained using the lasso, adaptive lasso, elastic net, and SSVS, using the same R functions and specifications used in our motivating example (described in 4.2). Because stepwise selection approaches have long been discouraged by social science methodologists (e.g., Henderson & Denison, 1989), even banned by editorial policy (Thompson, 1995), and to limit the scope of this paper, we do not include stepwise approaches for comparison in the present simulation study. We refer interested readers to many studies which have documented the performance of stepwise methods in comparison with other approaches (e.g., Hastie et al., 2020; Swartz et al., 2008)

#### Quantities Assessed

We calculated the mean estimates and inclusion rates for each method and condition averaging over true small, medium, large, and null effects. For SSVS, inclusion rates were calculated based on predictors with MIPs larger than .5. Within each replication and for each predictor we obtained the average coefficient estimate, including both zero and non-zero values. For lasso-type methods, predictor variables were coded as selected if they had a non-zero coefficient in the model produced for each replication, and the coefficients were also recorded. In addition to rates of inclusion by effect size, we compared overall model classification performance for the lasso and SSVS by varying the selection threshold ($\lambda$ for lasso and MIP for SSVS) and calculating sensitivity and specificity at each threshold. We then computed the area under the resulting ROC curve (AUC) for each method by sample size. We also examined the computational time for each method. In presenting our results we have emphasized graphical displays to reveal patterns observed for each method and factor, however complete quantitative results including observed absolute and relative bias are available in the online supplement.

## Simulation Results

We first describe the patterns of results for variable inclusion and coefficient estimates with uncorrelated predictors, followed by a description of the differences observed in the conditions with correlated predictors.

**Table 3:** Rates of inclusion by variable selection method, effect size, sample size, correlations among predictors, and pattern of true effects.

		*n * = 100						*n * = 400					
	Effect size	Lasso (min)%	Lasso (1SE)%	Lasso (BIC)%	Adapt. lasso %	Elastic net %	SSVS %	Lasso (min)%	Lasso (1SE)%	Lasso (BIC)%	Adapt. lasso %	Elastic net %	SSVS %
$\rho =$ 0	Null	35.8	13.5	24.1	7.7	36.8	2.8	36.4	6.9	19.9	2.8	38.4	1.4
	Small	92.8	78.6	.875	72.4	93.3	39.0	100	100	100	100	100	98.7
	Medium	100	100	100	100	100	99.9	100	100	100	100	100	99.9
	Large	100	100	100	100	100	100	100	100	100	100	100	100
$\rho =$ .4 mixed true	Null	35.4	15.9	24.7	8.0	36.7	3.5	36.0	10.6	22.4	3.4	37.3	1.5
	Small	91.6	77.6	85.0	68.3	91.7	36.8	100	100	100	100	100	98.2
	Medium	100	100	100	100	100	99.9	100	100	100	100	100	99.9
	Large	100	100	100	100	100	100	100	100	100	100	100	100
$\rho =$ .8 mixed true	Null	34.2	22.7	28.6	11.5	37.2	6.6	35.7	20.3	28.0	6.8	38.4	3.6
	Small	77.5	62.8	71.1	53.5	77.4	26.5	99.3	98.3	99.0	95.8	99.2	83.2
	Medium	100	100	100	99.8	100	96.5	100	100	100	100	100	99.8
	Large	100	100	100	100	100	99.9	100	100	100	100	100	99.9
$\rho =$ .4 clustered true	Null	21.6	4.2	12.2	4.2	22.3	2.0	20.8	1.0	10.7	0.4	21.6	1.0
	Small	96.4	92.9	95.4	77.5	96.6	34.8	100	100	100	99.2	100	91.9
	Medium	100	100	100	100	100	99.7	100	100	100	100	100	99.9
	Large	100	100	100	100	100	99.9	100	100	100	100	100	99.9
$\rho =$ .8 clustered true	Null	14.9	1.7	8.1	3.2	16.3	1.7	14.3	0.3	6.1	0.5	15.6	0.8
	Small	88.8	84.0	87.3	54.7	91.6	18.2	99.7	99.4	99.6	82.9	99.8	50.8
	Medium	100	100	100	99.9	100	91.6	100	100	100	100	100	99.6
	Large	100	100	100	100	100	99.7	100	100	100	100	100	99.7

BD, block diagonal covariance structure among sets of 5 predictors; mixed true, design with one true effect per block of correlated predictors; clustered true, design with all true effects in the first two blocks.

### Performance of Variable Selection Methods with Uncorrelated Predictors

#### Rates of Inclusion

The observed rates for variable inclusion for each method are shown in Table 3. With uncorrelated predictors, medium and large effects were selected in more than 99% of replications for all methods and for both sample sizes. In general the elastic net and lasso(min) resulted in the most predictors included, including false inclusion of about 36% of null effects for both small and large sample sizes. Fewer predictors were selected using the lasso(1SE) criterion, and the rate of false inclusion improved with sample size (13.5% for $n =$ 100 and 6.9% for $n =$ 400). The false inclusion rate for the lasso(BIC) solutions fell between the rate for lasso(1SE) and lasso(min) (24.1% for $n =$ 100 and 19.9% for $n =$ 400. Of the lasso methods, the adaptive lasso selected the fewest predictors. The adaptive lasso selected 3–8% of null effects, and 1.5$-$3.5% were included using SSVS. Differences in rates of inclusion were most apparent for small effects when the sample size was small ($n =$100): lasso(min) resulted in 98% of small predictors selected, while SSVS resulted in inclusion of 39% of small predictors. With the larger sample size of $n =$ 400, SSVS and all lasso methods selected > 98% of small effects. Because each replication included 50 predictors (10 true effects and 40 null effects), these rates of inclusion resulted in the largest models for lasso(min) and smallest using SSVS. On average 24 predictors were included using lasso(min) in both sample size conditions; SSVS resulted in an average of 7.5 predictors with $n =$ 100 and the true model size of 10 predictors with $n =$ 400.

In terms of overall classification performance across thresholds of $\lambda$, the average AUC for lasso was .95 for either sample size. Across MIP thresholds, the average AUC for SSVS was .98 for a sample size of 100 and 1.0 for a sample size of 400.

#### Coefficient Estimates

**Fig. 2:**
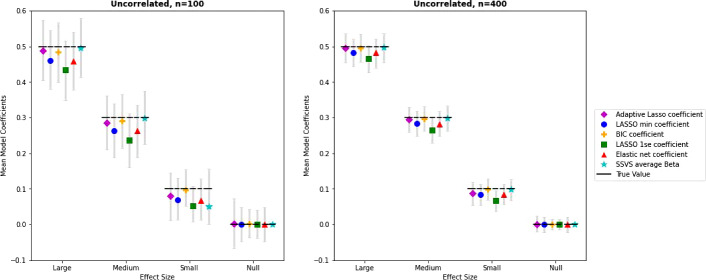
Average coefficient estimates by method, effect size, and sample size for conditions with uncorrelated predictors. *Note.* True parameter values are shown by the dashed horizontal line. Mean estimates are shown as symbols by each method with 95% intervals of observed estimates. SSVS average coefficient estimates are inclusive of both zero and non-zero values.

The coefficient estimates for the lasso methods and SSVS with uncorrelated predictors are presented in the first column of Fig. 2 along with 95% intervals of observed estimates. Interested readers can find complete tables with mean estimates, absolute and relative bias, and standard deviations of the estimates in the supplemental materials. Bias is illustrated in Fig. 2 by the deviation of the mean estimate for each method from the line denoting the true effect size. Lasso estimates were systematically biased towards zero; the absolute shrinkage is uniform for large and small coefficients and decreased with larger sample size (~3.7% for $n =$ 100 and ~1.7% for $n =$ 400 for the lasso(min)). However note this results in larger relative bias for smaller effects (e.g. 15.6% average relative bias for small effects versus 3.5% relative bias for large effects for $n =$ 400). The lasso (1SE) solution resulted in slightly more shrinkage. For large and medium effects, SSVS, lasso(BIC), and adaptive lasso estimates were nearly unbiased. For small effects, the average estimate from SSVS was shrunk towards zero when the sample size was small but nearly unbiased with larger *n*.

As shown by the intervals of observed estimates in Fig. 2, variability in estimates decreased with sample size and was similar across methods for large and medium sized effects. For small effects, variability was slightly higher for estimates from SSVS. The lasso solutions produced more variability in estimates for true null effects, with the adaptive lasso having the most variability. These differences in variability in estimates for null effects are related to the differences in rates of inclusion for the null predictors.

### Performance of Variable Selection Methods with Correlated Predictors

#### Rates of Inclusion with Mixed True Effects

For all methods, rates of inclusion for medium and large effects were almost identical in these conditions compared to the baseline condition with all uncorrelated predictors. For small effects and true zero effects, this pattern of correlations among true and null effects differed by variable selection method. Interestingly, the rate of false inclusion did not increase for the lasso(min) solution. False inclusion rates increased for the lasso(1SE) and lasso(BIC) solutions, for example the false inclusion rate with $\sigma = .4$ increased to 15.9% for lasso(1SE) for $n =$ 100 (10.6% for $n =$ 400). False inclusion rates increased less for adaptive lasso and remained the lowest for SSVS. The highest rate of false inclusion for SSVS was 6.6% for the condition with small *n* and high correlations among predictors. These conditions also resulted in lower rates of inclusion for small effects for all methods.

The AUC was highest in these conditions for SSVS: .97 with $n =$ 100 and 1.0 with $n =$ 400 for $\sigma =.4$. These values decreased to .93 and 1.0 for $\sigma =$ .8 with $n =$ 100 and $n =$ 400, respectively. For lasso the AUCs were .93 and .95 for $n =$ 100 and 400 with $\sigma =$ .4 and decreased with higher correlation among the predictors (.85 and .93 for $n =$ 100 and 400 with $\sigma =$ .8).

**Fig. 3:**
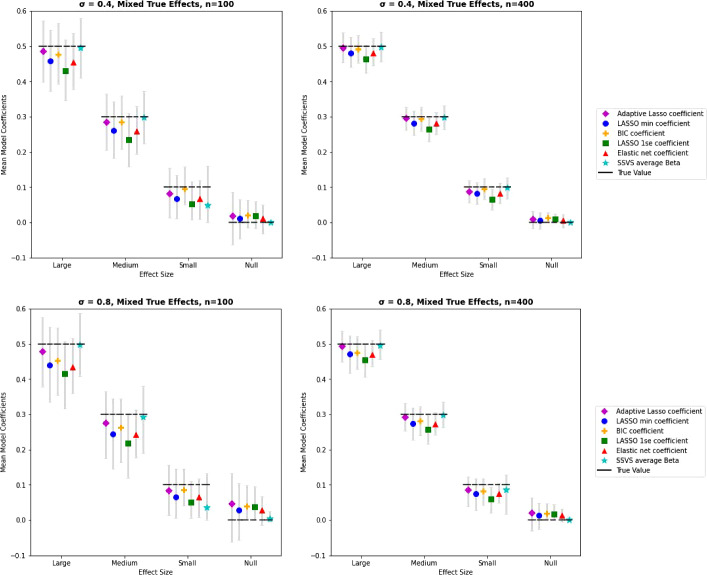
Average coefficient estimates by method, effect size, and sample size for conditions with correlated predictors, mixed true effects. *Note.* True parameter values are shown by the dashed horizontal line. Mean estimates are shown as symbols by each method with 95% intervals of observed estimates. SSVS average coefficient estimates are inclusive of both zero and non-zero values.

#### Coefficient Estimates

The correlation pattern in these conditions did not have a noticeable impact on estimate bias, as seen in Fig. 3. When the correlation among predictors was high, variability in estimates of true effects increased for all methods. Of the lasso methods, the variability in estimates remained highest for adaptive lasso, lowest for lasso(1SE), and lasso(min) falling in between. Estimates of true zero effects increased as the correlation among predictors increased in these conditions. Variability remained low for estimates of null effects produced by SSVS.

**Fig. 4:**
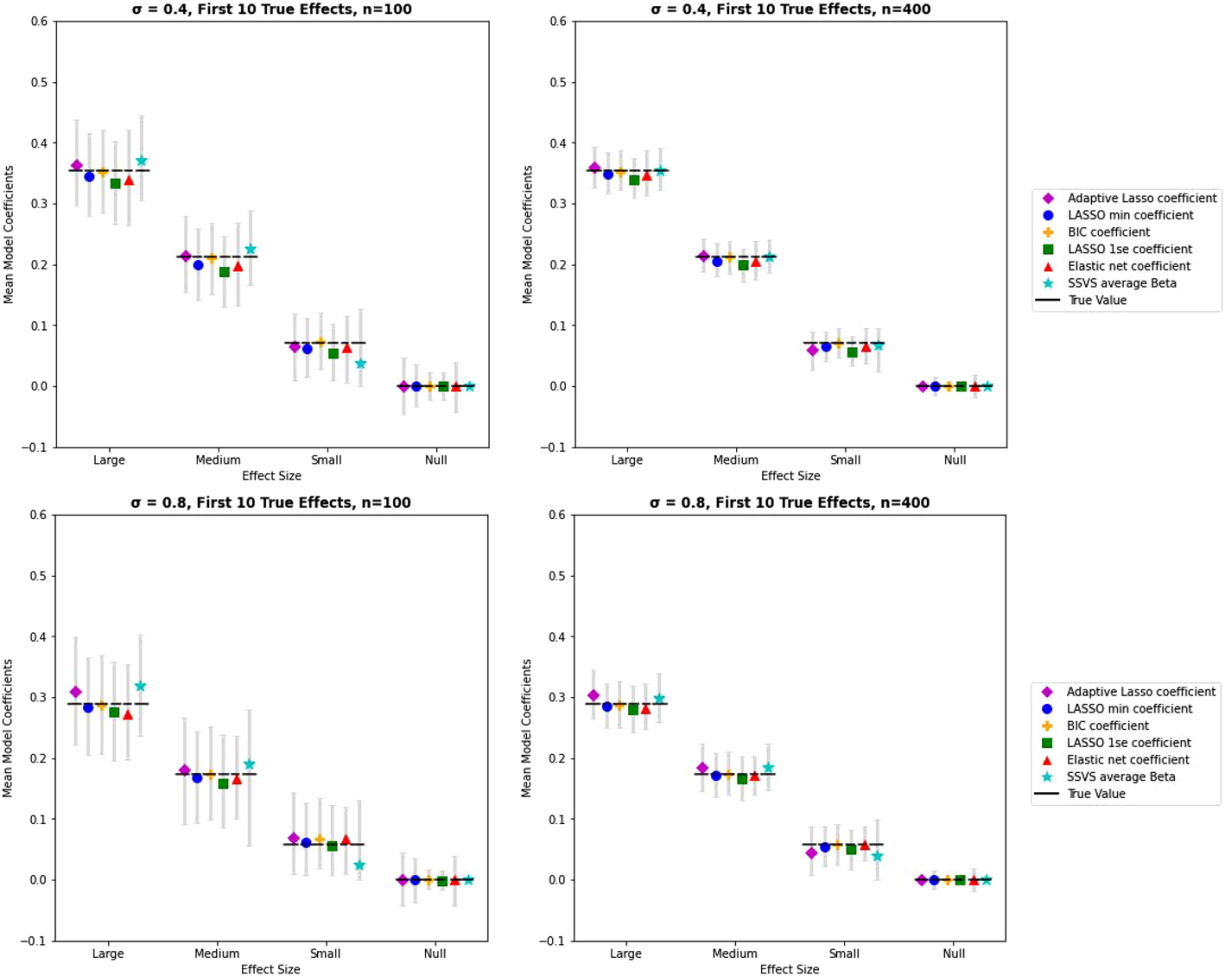
Average coefficient estimates by method, effect size, and sample size for conditions with correlated predictors, clustered true effects. *Note.* *Y*-axis scaling is equivalent to previous Figs. 2 and 3, however note that true effect size decreases as the correlation among true effects increases. True parameter values are shown by the dashed horizontal line. Mean estimates are shown as symbols by each method with 95% intervals of observed estimates. SSVS average coefficient estimates are inclusive of both zero and non-zero values.

#### Rates of Inclusion and Estimates for Clusters of Correlated True Effects

Figure 4 illustrates the pattern of results for coefficient estimate bias and variability for clusters of true effects. Because the true effects are smaller for correlated predictors, the estimated coefficients decreased as the correlation among true effects increased. The variability of the estimates also increased with the correlation among true effects. Similarly, rates of inclusion for small effects were lower, especially for SSVS. The rates of inclusion for null effects decreased as the correlation among true effects increased for lasso(1SE), lasso(min), lasso(BIC), and adaptive lasso. The correlation among true effects also corresponded to decreased variability in the coefficient estimates. The rate of inclusion for null effects remained low for SSVS.

The AUCs for SSVS at each sample size were virtually identical to the values with uncorrelated predictors (.98 and 1.0 for $n =$ 100 and 400 with $\sigma =$ .4;.97 and 1.0 for $n =$ 100 and 400 with $\sigma =$ .8). For lasso the AUCs were slightly lower (.96 and .93 for $n =$ 100 and 400 with $\sigma =$ .4) and decreased with higher correlation among the predictors (.93 and .91 for $n =$ 100 and 400 with $\sigma =$ .8).

### Computational Time

We assessed average computational time depending on sample size, from $n =$ 100 to $n =$ 800, and the correlation conditions presented above. Additionally, to assess the impact of increasing the number of candidate predictors, we benchmarked computational time with 25 versus 50 predictors. Average run times are using a personal computer (12 GB RAM, four core 2.7 GHz processor). Lasso solutions were produced using the extremely efficient LARS algorithm programmed into the glmnet package. For all sample sizes and conditions, run time for the lasso solutions was approximately .1 s. The adaptive lasso run time was slightly longer, about .8 s on average with 25 predictors and .9 s on average for 50 predictors, regardless of sample size and correlation structure. By contrast, SSVS was much slower, and computation time depended on both the sample size and number of predictors. For example, average run time for $n =$ 100 was 18 s with 25 predictors compared to 32 s with 50 predictors. Computational time also increased as sample size increased: 25 s on average for a sample size of 400 and 40 s with 800.

### Summary of Simulation Results

In general, the behavior of SSVS was most stable across conditions. For medium and large effects, or small effects with a large sample size, SSVS selected true effects and, when selected, estimated coefficients with minimal bias. SSVS resulted in the selection of null effects below 7% in all conditions. Computational time was an order of magnitude longer for SSVS compared to lasso methods, but not intractable for the conditions studied. As a Bayesian method which uses MCMC to summarize the posterior distribution, computational time for SSVS is justifiably longer. The algorithm used in the SSVSforPsych package is straightforward, but it has not been optimized for computational efficiency. More sophisticated algorithms can be used to improve efficiency (e.g. BoomSpikeSlab, Scott, 2018). Of the lasso methods, adaptive lasso resulted in the lowest false inclusion rates (between 0.4 and 11.5% in the conditions studied) and minimal bias for estimated true effects. However, estimates for false positive effects were more variable. The traditional lasso solution had high bias and high rates of inclusion of null effects. The lasso(1SE) solution improved the rates of inclusion for null effects, but increased bias in true effects.

## Conclusions and Future Directions

In many cases a research question can be specified and tested as a formal model, guided by theory and building on previous findings. In these cases a researcher’s substantive knowledge of the phenomenon under study can be more important than accepting any automated variable selection rule or procedure. Where theory or background information is insufficient to formulate this model a priori, researchers may wish to narrow down a candidate predictor set in a principled way. For this goal, classic techniques like stepwise regression methods are inadequate. Modern regularization methods such as the lasso are widely used in other fields, and there has been extensive recent development of research methods in psychology incorporating lasso regularization. However less attention has been paid to understanding how lasso regularization may perform in conditions commonly encountered in our field.

In this paper we compare the behavior of SSVS, a Bayesian variable selection method, to commonly used variations of the lasso. We found several advantages of SSVS relative to the lasso: a continuous measure of variable importance, very low rates of false inclusion, and relatively high rates of correct inclusion. We found that SSVS had a relatively low rate of inclusion for small effects when the sample size is small (39%, $n =$ 100), but the power to detect even small effects was high with a larger sample size (99%, $n = 400$). Lasso methods showed much higher rates of false inclusion, a stronger penalty on true effects, and dichotomously include or exclude predictors without a probabilistic estimate associated with variable inclusion. Of note, post-selection hypothesis tests have been recently developed for lasso methods (Tibshirani et al., 2014), though these methods are rarely used in practice. Future research should investigate and compare their performance with SSVS.

Although these results are not exhaustive, they provide a framework for understanding how SSVS is expected to perform and compare to lasso-type regression under a range of representative conditions. For researchers interested in performing variable selection and interpreting the importance of predictors, our results suggest that SSVS provides more useful and interpretable information than lasso methods. Factors varied in this study included sample size, effect size, and patterns of correlation among predictors and true effects. Several factors were not examined, but we can make some inferences about how our results may generalize. We do not include results varying the number of predictors, but in preliminary examinations we did not notice any substantive differences in results for 25 versus 50 predictors (conditions omitted for brevity but available upon request). We also did not systematically vary the prior inclusion probability, opting to use the default prior probability of .5 for all predictors and a diffuse prior for the (standardized) regression coefficients. This prior reflects a belief that 1/2 of the predictors should be included. In our simulations the true number of effects was 10 (1/5 of the predictors), and our results do not indicate that the prior used for SSVS unduly influenced the model size.

A comprehensive comparison of Bayesian and penalized likelihood variable selection approaches was outside the scope of this paper, though we hope that our review and simulation results can help create a framework for comparison. Though our example and simulation conditions are tailored to psychology, our results are also consistent with other findings in the literature. A recent study comparing a total of 21 different Bayesian and penalized regression methods across a wide range of real datasets

Several important future directions are needed in this area. Missing data is pervasive to research in our field, and unfortunately there is not currently an accepted way to jointly account for the uncertainty arising from missing data with the uncertainty of model selection (Jiang et al., 2015). Commonly, listwise deletion is used to arrive at a dataset with complete values, and this is a major limitation. As we found, more sophisticated algorithms (e.g. Scott, 2018) would be needed to perform SSVS with much larger datasets, while lasso methods are sufficiently scaled to large data problems. Another interesting development is the spike-and-slab lasso (Ročková & George, 2018), which integrates spike-and-slab methodology and penalized likelihood estimation to overcome biases in the lasso. We have limited our focused here to simple linear regression models, but methods are available for nested data (Müller et al., 2012) and categorical outcomes. Interactions among predictors can be crucial to understanding psychological phenomena and are central to prominent theoretical models such as diathesis-stress models (Zuckerman). Extensions to SSVS have been developed for selection with grouped predictors, such as main effects with interactions or multiple regressors for a categorical variable (Farcomeni, 2010). These extensions have not been disseminated to researchers in the behavioral sciences, and it is important to understand their performance in finite samples. Given the flexibility and advantages of a Bayesian variable selection framework, we hope to see these methods considered in psychological research.

## Supplementary Information

Below is the link to the electronic supplementary material.

Supplementary file 1 (pdf 93 KB)

## Footnotes

1 Tibshirani’s (1996) introduction of the lasso is the second most highly cited paper published in the prestigious Journal of the Royal Statistical Society, Series B (the third most cited is the elastic net extension of the lasso; Zou & Hastie, 2005).
