# Supplementary material for: Comparing Bayesian Variable Selection to Lasso Approaches for Applications in Psychology
Source: Psychometrika. 2023 May 23;88(3):1032–55. doi: 10.1007/s11336-023-09914-9 (PMC10202760; doi:10.1007/s11336-023-09914-9)
Supplement: Supplementary file 1 — (pdf 93 KB) [file 11336_2023_9914_MOESM1_ESM.pdf]

Table S1
Summaries of coefficient estimates by method, effect size, and sample size with uncorrelated predictors

Effect Size	True Val	Mean Est	Med Est	SD Est	Raw Bias	.05 Q Est	.95 Q Est
**N = 100, Uncorrelated Predictors**							
LASSO (min)							
Large	0.5	0.461	0.462	0.050	0.039	0.381	0.545
Medium	0.3	0.263	0.263	0.046	0.037	0.189	0.339
Small	0.1	0.069	0.067	0.035	0.031	0.014	0.130
Null	0.0	0.000	0.000	0.030	0.000	-0.048	0.048
LASSO (1SE)							
Large	0.5	0.434	0.436	0.049	0.066	0.349	0.517
Medium	0.3	0.236	0.236	0.047	0.064	0.160	0.312
Small	0.1	0.052	0.049	0.032	0.048	0.007	0.109
Null	0.0	0.000	0.000	0.025	0.000	-0.039	0.042
SSVS							
Large	0.5	0.496	0.498	0.051	0.004	0.414	0.579
Medium	0.3	0.298	0.297	0.046	0.002	0.225	0.375
Small	0.1	0.050	0.027	0.053	0.050	0.001	0.156
Null	0.0	0.000	0.000	0.006	0.000	-0.003	0.003
Adaptive LASSO							
Large	0.5	0.489	0.489	0.050	0.011	0.404	0.573
Medium	0.3	0.286	0.285	0.047	0.014	0.211	0.362
Small	0.1	0.081	0.081	0.040	0.019	0.011	0.145
Null	0.0	0.002	0.001	0.045	-0.002	-0.067	0.072
**N = 400, Uncorrelated Predictors**							
Effect Size	*True Val*	*Mean Est*	*Med Est*	*SD Est*	*Raw Bias*	*.05 Q Est*	*.95 Q Est*
LASSO (min)							
Large	0.5	0.483	0.484	0.024	0.017	0.443	0.521
Medium	0.3	0.283	0.282	0.021	0.017	0.248	0.318
Small	0.1	0.084	0.084	0.018	0.016	0.055	0.114
Null	0.0	0.000	0.000	0.013	0.000	-0.022	0.021
LASSO (1SE)							
Large	0.5	0.465	0.466	0.024	0.035	0.426	0.504
Medium	0.3	0.265	0.264	0.021	0.035	0.230	0.300
Small	0.1	0.067	0.066	0.018	0.033	0.037	0.096
Null	0.0	0.000	0.000	0.010	0.000	-0.015	0.016
SSVS							
Large	0.5	0.498	0.499	0.024	0.002	0.455	0.537
Medium	0.3	0.298	0.297	0.021	0.002	0.263	0.333
Small	0.1	0.099	0.099	0.019	0.001	0.068	0.127
Null	0.0	0.000	0.000	0.002	0.000	-0.001	0.001
Adaptive LASSO							
Large	0.5	0.496	0.497	0.024	0.004	0.454	0.536
Medium	0.3	0.295	0.295	0.022	0.005	0.260	0.330
Small	0.1	0.088	0.088	0.020	0.012	0.055	0.119
Null	0.0	0.001	0.001	0.016	-0.001	-0.021	0.025

Table S2

Summaries of coefficient estimates by method, effect size, and sample size with moderately correlated predictors, mixed true effects

**N = 100, Correlation = 0.40, Mixed True Effects**							
Effect Size	*True Val*	*Mean Est*	*Med Est*	*SD Est*	*Raw Bias*	*.05 Q Est*	*.95 Q Est*
LASSO (min)							
Large	0.5	0.458	0.458	0.054	0.042	0.372	0.546
Medium	0.3	0.261	0.260	0.048	0.039	0.182	0.342
Small	0.1	0.068	0.066	0.038	0.032	0.012	0.135
Null	0.0	0.011	0.011	0.033	-0.011	-0.046	0.065
LASSO (1SE)							
Large	0.5	0.430	0.430	0.052	0.070	0.346	0.518
Medium	0.3	0.234	0.234	0.047	0.066	0.158	0.310
Small	0.1	0.052	0.047	0.033	0.048	0.007	0.115
Null	0.0	0.018	0.016	0.024	-0.018	-0.017	0.060
SSVS							
Large	0.5	0.495	0.498	0.052	0.005	0.410	0.580
Medium	0.3	0.299	0.299	0.045	0.001	0.224	0.372
Small	0.1	0.048	0.025	0.054	0.052	0.001	0.161
Null	0.0	0.001	0.000	0.008	-0.001	-0.003	0.006
Adaptive LASSO							
Large	0.5	0.486	0.488	0.053	0.014	0.398	0.572
Medium	0.3	0.285	0.285	0.047	0.015	0.205	0.365
Small	0.1	0.082	0.082	0.042	0.018	0.013	0.154
Null	0.0	0.018	0.022	0.048	-0.018	-0.064	0.086
**N = 400, Correlation = 0.40, Mixed True Effects**							
Effect Size	*True Val*	*Mean Est*	*Med Est*	*SD Est*	*Raw Bias*	*.05 Q Est*	*.95 Q Est*
LASSO (min)							
Large	0.5	0.481	0.481	0.025	0.019	0.441	0.525
Medium	0.3	0.282	0.282	0.021	0.018	0.247	0.316
Small	0.1	0.083	0.082	0.019	0.017	0.052	0.113
Null	0.0	0.005	0.005	0.014	-0.005	-0.019	0.029
LASSO (1SE)							
Large	0.5	0.464	0.463	0.024	0.036	0.425	0.503
Medium	0.3	0.265	0.265	0.021	0.035	0.230	0.298
Small	0.1	0.065	0.065	0.018	0.035	0.036	0.095
Null	0.0	0.009	0.007	0.009	-0.009	0.000	0.025
SSVS							
Large	0.5	0.498	0.497	0.025	0.002	0.457	0.540
Medium	0.3	0.299	0.299	0.021	0.001	0.265	0.332
Small	0.1	0.099	0.099	0.019	0.001	0.067	0.127
Null	0.0	0.000	0.000	0.002	0.000	-0.001	0.001
Adaptive LASSO							
Large	0.5	0.496	0.495	0.025	0.004	0.455	0.538
Medium	0.3	0.296	0.296	0.021	0.004	0.262	0.328
Small	0.1	0.088	0.089	0.020	0.012	0.056	0.120
Null	0.0	0.009	0.009	0.018	-0.009	-0.017	0.032

Table S3

Summaries of coefficient estimates by method, effect size, and sample size with highly correlated predictors, mixed true effects

**N = 100, Correlation = 0.80, Mixed True Effects**							
Effect Size	*True Val*	*Mean Est*	*Med Est*	*SD Est*	*Raw Bias*	*.05 Q Est*	*.95 Q Est*
LASSO (min)							
Large	0.5	0.440	0.440	0.065	0.060	0.336	0.547
Medium	0.3	0.245	0.246	0.062	0.055	0.145	0.344
Small	0.1	0.065	0.061	0.044	0.035	0.006	0.145
Null	0.0	0.029	0.027	0.048	-0.029	-0.055	0.104
LASSO (1SE)							
Large	0.5	0.415	0.417	0.059	0.085	0.316	0.507
Medium	0.3	0.219	0.222	0.056	0.081	0.120	0.302
Small	0.1	0.051	0.047	0.033	0.049	0.005	0.110
Null	0.0	0.037	0.030	0.032	-0.037	0.001	0.096
SSVS							
Large	0.5	0.497	0.496	0.055	0.003	0.408	0.586
Medium	0.3	0.292	0.297	0.063	0.008	0.190	0.381
Small	0.1	0.036	0.016	0.045	0.064	0.001	0.133
Null	0.0	0.004	0.001	0.017	-0.004	-0.006	0.024
Adaptive LASSO							
Large	0.5	0.479	0.480	0.060	0.021	0.378	0.576
Medium	0.3	0.275	0.279	0.060	0.025	0.175	0.365
Small	0.1	0.084	0.083	0.045	0.016	0.014	0.157
Null	0.0	0.047	0.051	0.062	-0.047	-0.061	0.132
**N = 400, Correlation = 0.80, Mixed True Effects**							
Effect Size	*True Val*	*Mean Est*	*Med Est*	*SD Est*	*Raw Bias*	*.05 Q Est*	*.95 Q Est*
LASSO (min)							
Large	0.5	0.472	0.472	0.032	0.028	0.417	0.523
Medium	0.3	0.274	0.273	0.029	0.026	0.228	0.319
Small	0.1	0.075	0.075	0.027	0.025	0.029	0.117
Null	0.0	0.013	0.013	0.022	-0.013	-0.026	0.049
LASSO (1SE)							
Large	0.5	0.455	0.456	0.028	0.045	0.407	0.500
Medium	0.3	0.257	0.257	0.025	0.043	0.216	0.298
Small	0.1	0.059	0.060	0.022	0.041	0.020	0.094
Null	0.0	0.017	0.014	0.014	-0.017	0.001	0.045
SSVS							
Large	0.5	0.496	0.496	0.026	0.004	0.456	0.540
Medium	0.3	0.298	0.297	0.022	0.002	0.263	0.335
Small	0.1	0.085	0.091	0.033	0.015	0.017	0.129
Null	0.0	0.001	0.000	0.007	-0.001	-0.002	0.009
Adaptive LASSO							
Large	0.5	0.493	0.493	0.027	0.007	0.449	0.536
Medium	0.3	0.293	0.293	0.024	0.007	0.254	0.332
Small	0.1	0.085	0.087	0.026	0.015	0.039	0.123
Null	0.0	0.021	0.022	0.031	-0.021	-0.030	0.064

Table S4

Summaries of coefficient estimates by method, effect size, and sample size with moderately correlated predictors, grouped true effects

**N = 100, Correlation = 0.40, First 10 True Effects**							
Effect Size	*True Val*	*Mean Est*	*Med Est*	*SD Est*	*Raw Bias*	*.05 Q Est*	*.95 Q Est*
LASSO (min)							
Large	0.35	0.344	0.343	0.041	0.009	0.280	0.415
Medium	0.21	0.199	0.199	0.036	0.012	0.142	0.259
Small	0.07	0.062	0.061	0.030	0.008	0.015	0.112
Null	0.00	0.000	0.000	0.021	0.000	-0.034	0.035
LASSO (1SE)							
Large	0.35	0.333	0.332	0.041	0.021	0.266	0.402
Medium	0.21	0.188	0.188	0.036	0.024	0.130	0.246
Small	0.07	0.054	0.052	0.029	0.017	0.010	0.103
Null	0.00	0.000	0.001	0.016	0.000	-0.023	0.022
SSVS							
Large	0.35	0.370	0.368	0.043	-0.017	0.305	0.445
Medium	0.21	0.226	0.225	0.038	-0.014	0.166	0.288
Small	0.07	0.037	0.016	0.044	0.033	0.000	0.126
Null	0.00	0.000	0.000	0.003	0.000	-0.001	0.001
Adaptive LASSO							
Large	0.35	0.363	0.362	0.043	-0.010	0.297	0.437
Medium	0.21	0.214	0.213	0.038	-0.002	0.154	0.280
Small	0.07	0.066	0.065	0.033	0.005	0.010	0.120
Null	0.00	0.000	0.001	0.032	0.000	-0.047	0.047
**N = 400, Correlation = 0.40, First 10 True Effects**							
Effect Size	*True Val*	*Mean Est*	*Med Est*	*SD Est*	*Raw Bias*	*.05 Q Est*	*.95 Q Est*
LASSO (min)							
Large	0.35	0.348	0.347	0.020	0.005	0.317	0.384
Medium	0.21	0.206	0.206	0.016	0.005	0.180	0.234
Small	0.07	0.065	0.065	0.015	0.006	0.042	0.089
Null	0.00	0.000	0.000	0.009	0.000	-0.015	0.015
LASSO (1SE)							
Large	0.35	0.340	0.339	0.020	0.013	0.309	0.374
Medium	0.21	0.199	0.198	0.017	0.013	0.172	0.226
Small	0.07	0.057	0.057	0.015	0.013	0.034	0.082
Null	0.00	0.000	0.000	0.006	0.000	-0.006	0.006
SSVS							
Large	0.35	0.355	0.354	0.020	-0.002	0.323	0.391
Medium	0.21	0.213	0.213	0.017	-0.001	0.187	0.241
Small	0.07	0.067	0.070	0.021	0.003	0.024	0.095
Null	0.00	0.000	0.000	0.001	0.000	0.000	0.000
Adaptive LASSO							
Large	0.35	0.359	0.358	0.020	-0.006	0.326	0.394
Medium	0.21	0.215	0.214	0.017	-0.003	0.188	0.243
Small	0.07	0.059	0.059	0.019	0.012	0.027	0.089
Null	0.00	0.000	0.000	0.004	0.000	-0.004	0.003

Table S5

Summaries of coefficient estimates by method, effect size, and sample size with highly correlated predictors, grouped true effects

**N = 100, Correlation = 0.80, First 10 True Effects**							
Effect Size	*True Val*	*Mean Est*	*Med Est*	*SD Est*	*Raw Bias*	*.05 Q Est*	*.95 Q Est*
LASSO (min)							
Large	0.29	0.284	0.281	0.047	0.005	0.205	0.366
Medium	0.17	0.168	0.167	0.045	0.006	0.094	0.244
Small	0.06	0.061	0.057	0.037	-0.003	0.008	0.127
Null	0.00	0.000	0.000	0.023	0.000	-0.037	0.036
LASSO (1SE)							
Large	0.29	0.276	0.275	0.047	0.013	0.196	0.357
Medium	0.17	0.159	0.159	0.045	0.014	0.085	0.238
Small	0.06	0.057	0.052	0.036	0.000	0.008	0.124
Null	0.00	-0.001	-0.001	0.013	0.001	-0.016	0.016
SSVS							
Large	0.29	0.319	0.318	0.050	-0.030	0.237	0.403
Medium	0.17	0.190	0.199	0.063	-0.017	0.057	0.279
Small	0.06	0.024	0.005	0.043	0.034	0.000	0.131
Null	0.00	0.000	0.000	0.003	0.000	-0.001	0.001
Adaptive LASSO							
Large	0.29	0.309	0.306	0.052	-0.020	0.222	0.399
Medium	0.17	0.180	0.181	0.052	-0.007	0.092	0.267
Small	0.06	0.070	0.067	0.041	-0.013	0.009	0.144
Null	0.00	0.000	0.000	0.031	0.000	-0.043	0.044
**N = 400, Correlation = 0.80, First 10 True Effects**							
Effect Size	*True Val*	*Mean Est*	*Med Est*	*SD Est*	*Raw Bias*	*.05 Q Est*	*.95 Q Est*
LASSO (min)							
Large	0.29	0.286	0.286	0.023	0.003	0.249	0.323
Medium	0.17	0.171	0.171	0.021	0.002	0.137	0.208
Small	0.06	0.055	0.056	0.020	0.002	0.023	0.088
Null	0.00	0.000	0.000	0.010	0.000	-0.015	0.015
LASSO (1SE)							
Large	0.29	0.280	0.280	0.023	0.009	0.242	0.318
Medium	0.17	0.166	0.166	0.021	0.007	0.131	0.203
Small	0.06	0.050	0.050	0.020	0.008	0.017	0.083
Null	0.00	0.001	0.001	0.002	-0.001	-0.001	0.002
SSVS							
Large	0.29	0.299	0.300	0.024	-0.010	0.260	0.339
Medium	0.17	0.185	0.185	0.022	-0.012	0.148	0.223
Small	0.06	0.040	0.033	0.034	0.018	0.001	0.098
Null	0.00	0.000	0.000	0.001	0.000	0.000	0.000
Adaptive LASSO							
Large	0.29	0.304	0.303	0.024	-0.015	0.264	0.344
Medium	0.17	0.184	0.184	0.023	-0.011	0.146	0.223
Small	0.06	0.045	0.044	0.025	0.013	0.007	0.087
Null	0.00	0.000	0.000	0.006	0.000	-0.005	0.005
